# Carotid plaque burden and hemodynamic alterations are associated with domain-specific cognitive impairment in community-dwelling older adults

**DOI:** 10.1038/s41598-026-66871-w

**Published:** 2026-08-22

**Authors:** Ahmed Elhfnawy, Mohamed Saud Kishk, Hany El Deeb, Jaidaa Mekky

**Affiliations:** Department of Neurology, Faculty of Medicine, Alexandria University, Alexandria, Egypt

**Keywords:** Carotid atherosclerosis, Mild cognitive impairment, Plaque score, Resistive index, Montreal cognitive assessment, Biomarkers, Diseases, Medical research, Neurology, Neuroscience

## Abstract

Carotid atherosclerosis has been linked to cognitive decline, but evidence regarding its association with domain-specific cognitive performance in understudied non-Western populations remains limited. We investigated the associations of carotid plaque burden and hemodynamic markers with cognitive impairment in 100 community-dwelling Egyptian adults aged ≥ 60 years without prior neurological disease. Carotid plaque burden was quantified by duplex ultrasound using plaque score, and cognition was assessed using the Montreal Cognitive Assessment (MoCA). Sixty-one Participants with a MoCA score ≤ 25 were classified as having possible mild cognitive impairment (MCI). Carotid plaques were detected in 53 participants, and plaque scores were higher in participants with possible MCI (*p* = 0.035). Internal carotid artery resistive index (ICA RI) was also higher (*p* = 0.011) among participants with possible MCI. In two separate multivariable models, plaque score (OR 1.41, 95% CI 1.06–1.86; *p* = 0.020) and ICA RI (OR 1.87, 95% CI 1.01–3.46; *p* = 0.046) were independently associated with possible MCI. Lower educational attainment showed the strongest association in both models. Plaque burden was most strongly associated with visuospatial and language performance. This cross-sectional study supports further longitudinal studies to determine whether carotid ultrasound markers predict subsequent cognitive decline.

## Introduction

Carotid atherosclerosis is a major public health concern, with high prevalence among elderly adults^1^. Beyond its role in stroke and cardiovascular disease, carotid plaque burden is linked to cognitive decline, even without overt cerebrovascular events^2^. Mild cognitive impairment (MCI), often preceding dementia, affects many older adults^3^, with dementia affecting tens of millions worldwide, particularly in low- and middle-income countries^4^. Many cross-sectional studies and meta-analyses indicate that greater carotid intima-media thickness (IMT) or plaque burden is associated with poorer global cognition and impairments in domains such as executive function and processing speed. However, longitudinal evidence remains heterogeneous: while some cohorts identify baseline IMT or composite plaque indices as predictors of subsequent cognitive decline, others report that plaque presence or area is no longer independently associated after adjustment for age, education, and vascular risk factors^5,6,7^. Notably, data from multiethnic and non-Western populations remain limited, restricting the generalizability of existing findings. This pilot cross-sectional study examines whether extracranial carotid plaque burden and hemodynamic markers are associated with global and domain-specific cognitive performance in community-dwelling older adults, adjusting for demographic and vascular risk factors.

## Methods

### Study design and population

This cross-sectional study enrolled 100 community-dwelling adults aged ≥ 60 years without previous stroke, dementia, or other neurological disease. Inclusion criteria were: (1) age ≥ 60 years, having ≥ 1 vascular risk factor, (2) absence of prior neurological disease, dementia, or acute cerebrovascular events (stroke or transient ischemic attack) and (3) absence of depression. Data were collected from July 2024 to August 2025. This study was conducted as an exploratory pilot cross‑sectional study, so the sample size of participants was determined by feasibility of eligible participants recruited during the study period. Written informed consent was obtained, and the study was approved by the Institutional Ethics Committee of Alexandria University Faculty of Medicine (Ethics no. 0108434), in accordance with the Declaration of Helsinki.

### Clinical assessment

Demographics (age, sex, body mass index, education: low ≤ 9 years, intermediate > 9–12 years, high > 12 years)^5^ and vascular risk factors (hypertension; defined as systolic blood pressure ≥ 140 mmHg, diastolic blood pressure ≥ 90 mmHg, or current use of antihypertensive medication, in accordance with the European Society of Hypertension (ESH) guidelines^6^; diabetes: fasting glucose ≥ 126 mg/dL, 2 h postprandial ≥ 200 mg/dL, or antidiabetics; smoking: non, ex [≥ 12 months], current; history of ischemic heart disease) were recorded.

### Ultrasound examination

Carotid duplex ultrasonography was performed by a single examiner using a Philips ClearVue 350 ultrasound system (Philips HealthCare, Best, The Netherlands) with a 4–12 MHz linear array transducer. The examiner was blinded to cognitive status. IMT was measured at the far wall of the distal 10 mm of each common carotid artery (CCA) in the longitudinal plane at the site of maximal thickness. IMT values ≥ 1 mm were classified as increased. Plaques were defined as focal echogenic structures or wall thickening ≥ 1.5 mm^7^. Stenosis severity was assessed using velocity criteria per the North American Symptomatic Carotid Endarterectomy Trial (NASCET) classification^8^. The bilateral carotid arterial tree was evaluated at four segments: (CCA), carotid bifurcation, internal carotid artery (ICA) and external carotid artery (ECA). Carotid plaque score quantified cumulative atherosclerotic burden across the four segments bilaterally. Plaque thickness ≥ 1.5 mm, ≥ 2.5 mm, and ≥ 3.5 mm were scored 1, 2, and 3 points, respectively; total plaque score ranged 0–24^7^. The internal carotid artery resistive index (ICA RI) was calculated as: RI = (peak systolic velocity − end-diastolic velocity)/peak systolic velocity^9^. For each participant, an ICA RI average was computed by mathematically averaging the bilateral (right and left) ICA RI measurements.

### Cognitive assessment

Global cognitive function was assessed using validated Arabic versions of the Montreal Cognitive Assessment (MoCA, version 8.3) and the Montreal Cognitive Assessment-Basic (MoCA-Basic). The MoCA-Basic was administered to participants with limited educational attainment or illiteracy, whereas the standard MoCA was administered to the remaining participants. The MoCA-Basic was administered and scored according to the official instructions, including the recommended education adjustments where applicable (+ 1 point for participants with ≤ 4 years of education and + 1 point for illiterate participants, if the total score was < 30). Both instruments generate total scores on a 30-point scale and assess comparable cognitive domains^10^. Participants were classified as possible MCI, if they had a MoCA score of ≤ 25; applied to both instruments for consistency across the study^11^. Because both MoCA and MoCA-Basic are screening instruments rather than diagnostic tools, this classification does not represent a clinically confirmed diagnosis of MCI.

### Statistical analysis

Continuous variables were summarized as median (interquartile range), categorical variables as counts and percentages. Mann-Whitney U and Kruskal-Wallis tests compared continuous variables between groups. Spearman correlation assessed associations between carotid measures and cognitive scores. Univariate logistic regression identified potential predictors of MCI; variables associated with the outcome of p value < 0.10 in univariate logistic regression were considered for inclusion in multivariable logistic regression models. Age and sex were retained in all multivariable models irrespective of their univariate significance because of their established clinical relevance as potential confounders. Two separate multivariable models were constructed: Model 1 included age, sex, educational level, and carotid plaque score, whereas Model 2 included age, sex, educational level, and ICA RI. Plaque score and ICA RI were analyzed in separate models to evaluate their independent associations with cognitive impairment and to avoid potential overlap between structural and hemodynamic carotid measures. Other risk factors as Hypertension and diabetes mellitus were not entered into the multivariable models because they did not meet the predefined inclusion criterion (*p* < 0.10) in univariate analysis. Model fit was evaluated with the Hosmer-Lemeshow test and multicollinearity with variance inflation factors. Analyses were conducted using SPSS version 27 (IBM Corp., Armonk, NY, USA).

## Results

**Fig. 1:**
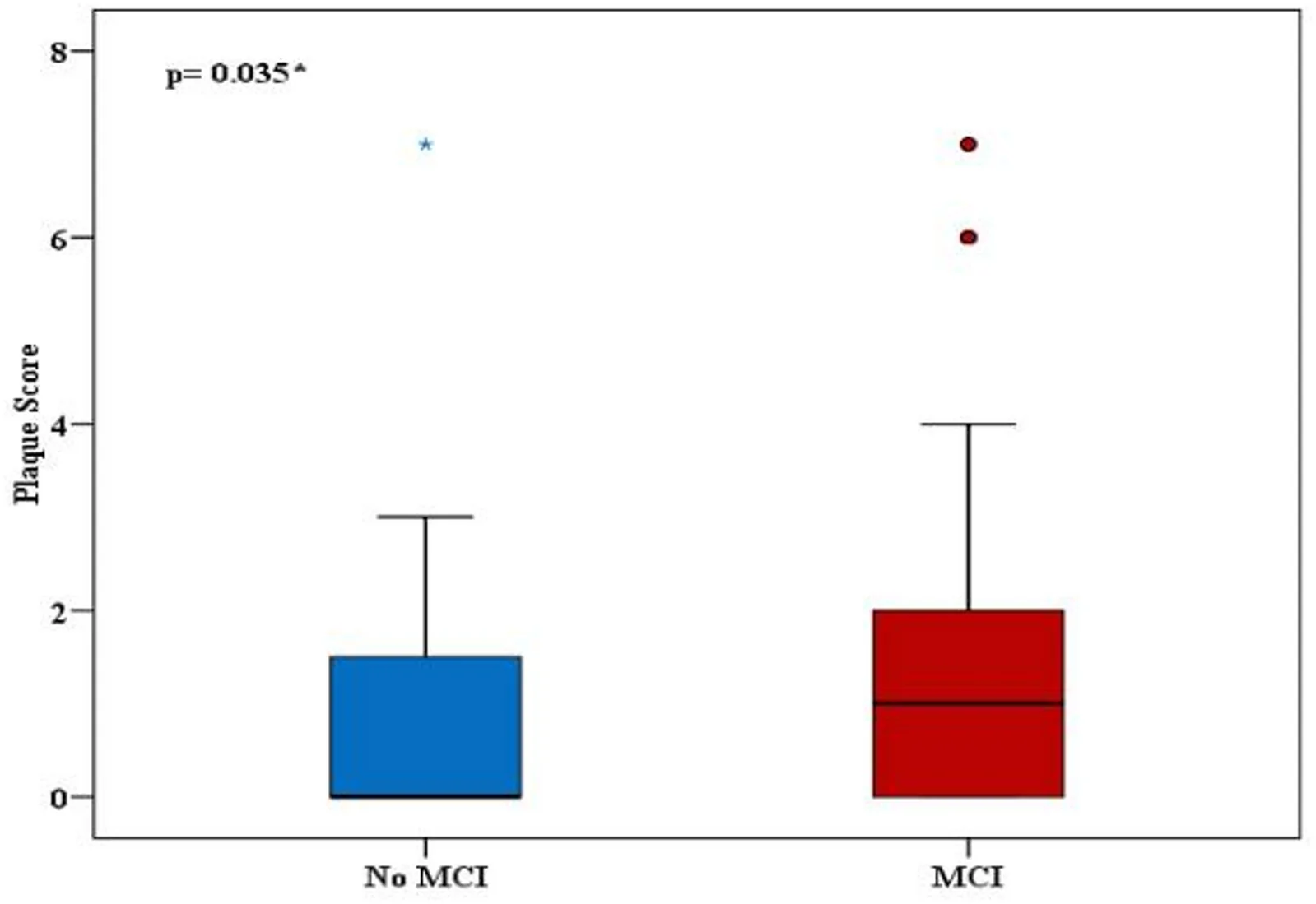
Relation between possible mild cognitive impairment (MoCA ≤ 25) (MCI) with Carotid Plaque Score (*n* = 100).

**Fig. 2:**
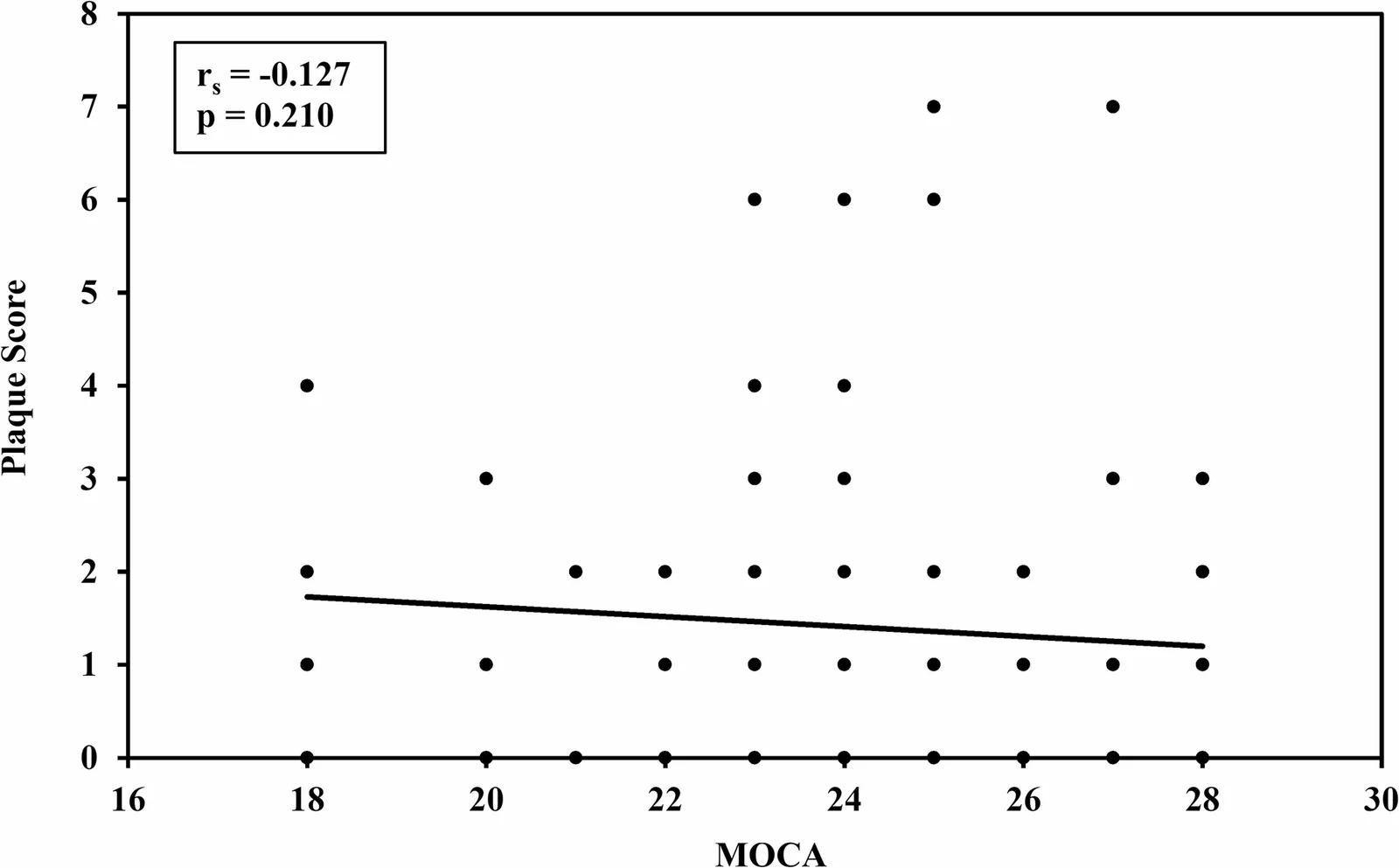
Correlation between Carotid Plaque Score with MOCA (*n* = 100).

**Fig. 3:**
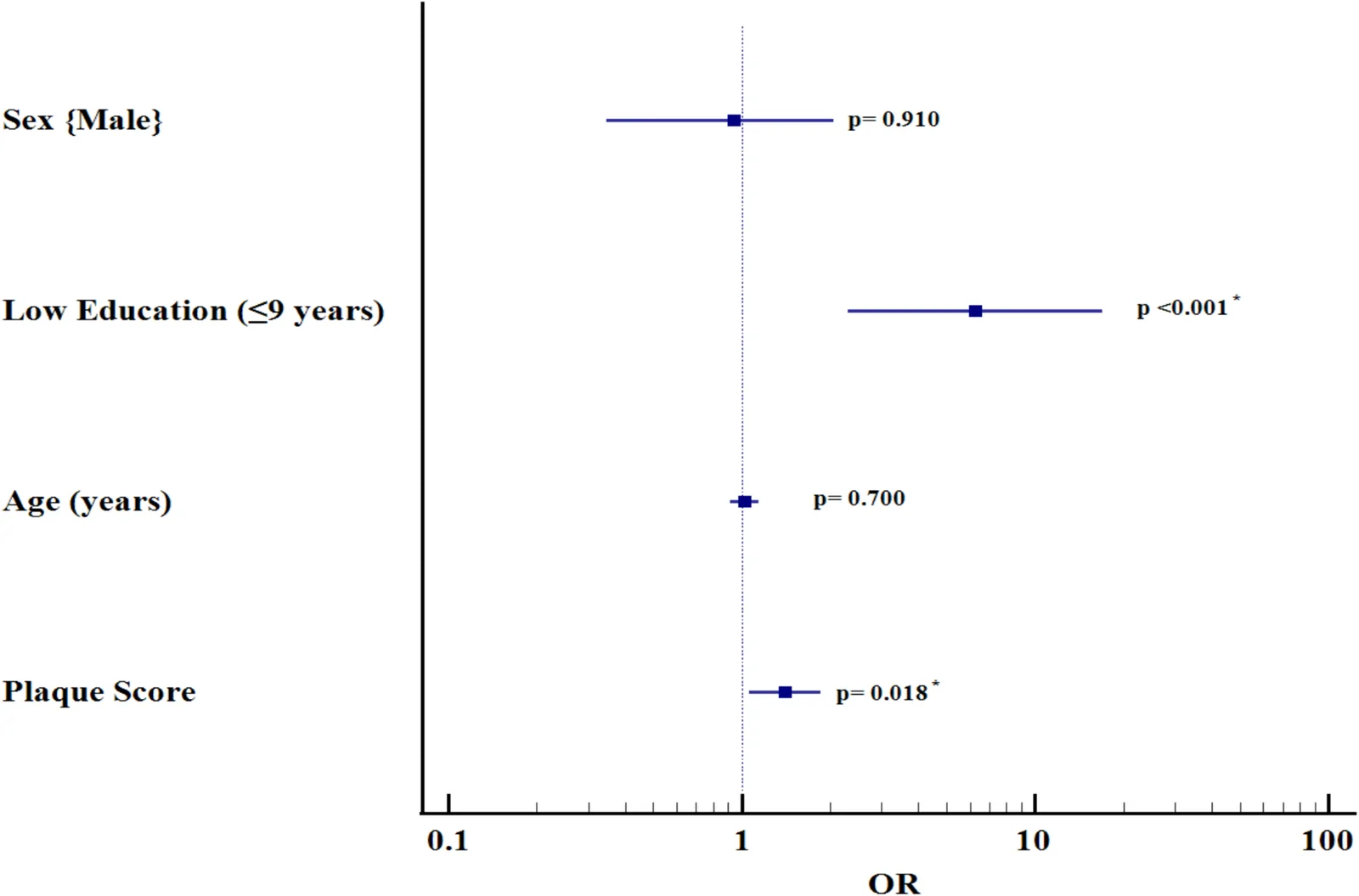
Model 1#Multivariate logistic regression analysis for baseline characteristics affecting possible mild cognitive impairment (MoCA ≤ 25) CI (no. of CI = 61 vs. no. of No CI = 39).

The study included 100 non-stroke adults from the general Egyptian population (median age 64 years, IQR 62–68; 58% male). Baseline characteristics of the overall study population are presented in Table 1. Based on MoCA screening, 61 participants (61%) were classified as possible MCI (MoCA ≤ 25), while 39 (39%) had normal cognitive screening (MoCA 26–30) (Table 2). Higher education (> 9 years) was more frequent in cognitively normal participants (69.2% vs. 29.5%, *p* < 0.001). Increased CCA IMT (≥ 1 mm) was present in 65 participants (65%), and carotid plaques were detected in 53 (53%). Significant ICA stenosis (≥ 50%) was observed in two participants (2%), both on the right side, one of them received carotid artery stenting. Plaques were most frequently located at the carotid bifurcation (32% right, 30% left), followed by the ICA, CCA, and ECA. Participants with possible MCI had a higher total plaque score than cognitively normal participants (median 1.0 [IQR 0.0–2.0] vs. 0.0 [IQR 0.0–1.5], *p* = 0.04; Fig. 1). Participantswith possible MCI had a higher median ICA RI (0.63 [IQR 0.57–0.71]) compared with cognitively normal participants (0.59 [IQR 0.53–0.62], *p* = 0.01). Age correlated positively with plaque score (Spearman ρ = 0.220, *p* = 0.03). Participants with plaques were older than those without plaques (median 66.0 vs. 64.0 years, *p* = 0.04). Age correlated inversely with global MoCA score (ρ=−0.297, *p* = 0.003) and with visuospatial, attention, recall, and orientation domains (Table 3). Plaque score correlated inversely with visuospatial (ρ=−0.26, *p* = 0.009) and language performance [Sentence repetition and verbal fluency] (ρ=−0.30, *p* = 0.003), but not with global MoCA score (ρ=−0.127, *p* = 0.210; Fig. 2). The association between carotid plaque presence and possible MCI did not reach statistical significance (χ²=3.68, *p* = 0.055; OR 2.22, 95% CI 0.98–5.03).Educational attainment showed strong associations with cognition. Median global MoCA scores increased across education levels (24, 25, and 26 for low, intermediate, and high education, respectively; *p* < 0.001). Lower education was associated with poorer visuospatial and attention performance (both *p* < 0.001) and lower abstraction scores (*p* = 0.046). Univariate logistic regression was performed including age, sex, hypertension, diabetes mellitus, educational attainment, plaque score, and ICA RI . Three variables were significantly associated with possible MCI. Low educational attainment showed the strongest association, with 5.4-fold higher odds of possible MCI compared to high education (OR = 5.38, 95% CI 2.24–12.89, *p* < 0.001). Carotid plaque score was also significantly associated, with each unit increase associated with 32.4% increased odds of possible MCI (OR = 1.32, 95% CI 1.02–1.72, *p* = 0.04). ICA RI also demonstrated a univariate significant association, with each 0.1-unit increase associated with 86.3% increased odds of possible MCI (OR = 1.863, 95% CI 1.11–3.14, *p* = 0.02). Sex, age, hypertension, and diabetes were not significant.Two multivariable logistic regression models were constructed. Variables with *p* < 0.10 in univariate analysis were considered for inclusion, while age and sex were retained in both models because of their clinical relevance. Plaque score and ICA RI were modeled separately because they represent related structural and hemodynamic carotid measures. In model 1 (Table 4), plaque score (OR 1.41, 95% CI 1.06–1.86, *p* = 0.02) and lower educational attainment (OR 6.26, 95% CI 2.30–17.02, *p* < 0.001) remained independently associated with possible MCI (Fig. 3). In Model 2, ICA RI (OR 1.87, 95% CI 1.01–3.46, *p* = 0.046) and lower educational attainment (OR 5.54, 95% CI 2.08–14.81, *p* = 0.001) remained significant.

**Table 1:** Distribution of the studied cases according to baseline characteristics (*n* = 100).

Baseline characteristics	
Male sex n (%)	58 (58%)
Education n (%)	
Low	55 (55%)
Intermediate	14 (14%)
High	31(31%)
Age (years)	
Min. – Max.	60.0–78.0
Median (IQR)	64.0 (62.0–68.0)
Obesity n (%)	37 (37%)
BMI (kg/m^2^) n (%)	
Normal weight (< 25)	32 (32%)
Overweight (25–29)	31 (31%)
Obese (≥ 30)	37 (37%)
Median (IQR)	27.0 (24.0–31.0)
Hypertension n (%)	52 (52%)
Diabetes mellitus n (%)	26 (26%)
Ischemic heart disease n (%)	29 (29%)
Smoking n (%)	
Non smoker	73 (73%)
Ex-smoker	13 (13%)
Smoker	14 (14%)
Plaque score [median (IQR)]	0.50 (0.0–2.0)
Increased CCA IMT n (%)	65 (65.0%)
Atherosclerotic plaque presence n (%)	53 (53.0%)
MOCA [Median (IQR)]	25.0 (23.0–26.0)
Possible MCI group n (%)	61 (61.0%)

BMI: body mass index, CCA: common carotid artery, IMT: intima-media-thickness, IQR: Inter quartile range, MoCA: Montreal cognitive assessment, MCI: Mild Cognitive impairment.

**Table 2:** Baseline characteristics of participants with and without possible mild cognitive impairment (Possible MCI) (*n* = 100).

Baseline characteristics	No Possible MCI (*n* = 39)	Possible MCI (*n* = 61)	*p*
Male sex n (%)	25 (64.1%)	33 (54.1%)	0.323
Education n (%)			
Low	12 (30.8%)	43 (70.5%)	< 0.001*
Intermediate	3 (7.7%)	11 (18.0%)	
High	24 (61.5%)	7 (11.5%)	
Age (years)			
Min.–Max.	60.0–78.0	60.0–75.0	0.070
Median (IQR)	63.0 (61.0–65.5)	65.0 (62.0–70.0)	
BMI (kg/m^2^)			
Median (IQR)	27.0 (24.0–31.0)	27.0 (24.0–31.0)	0.611
Hypertension n (%)	17 (43.6%)	35 (57.4%)	0.178
Diabetes mellitus n (%)	8 (20.5%)	18 (29.5%)	0.317
Smoking n (%)			
Non-smoker	30 (76.9%)	43 (70.5%)	0.753
Ex-smoker	4 (10.3%)	9 (14.8%)	
Smoker	5 (12.8%)	9 (14.8%)	
Plaque score [median (IQR)]	0.0 (0.0–1.50)	1.0 (0.0–2.0)	0.035*
ICA resistive index [median (IQR)]	0.59 (0.53–0.62)	0.63 (0.57–0.71)	0.011*

BMI: Body Mass Index, ICA: Internal Carotid Artery, IQR: Inter quartile range, MCI:Mild Cognitive Impairment, MoCA screening cut-off for possible MCI: ≤25.

U: Mann Whitney test χ^2^: Chi square test.

p: p value for Relation between Possible MCI with Baseline characteristics.

*: Statistically significant at *p* ≤ 0.05.

**Table 3:** Correlation between age and Carotid Plaque Score and montreal cognitive assessment [MOCA] (*n* = 100).

	Age (years)	
	*r*_s_	*p*
Plaque score	0.220*	0.028*
MOCA	− 0.297*	0.003*
Visuospatial	− 0.248*	0.013*
Attention	− 0.223*	0.026*
Language	− 0.188	0.062
Abstraction	0.037	0.716
Recall	− 0.270*	0.007*
Orientation	− 0.268*	0.007*

r_s_: Spearman coefficient MOCA: montreal cognitive assessment.

*: Statistically significant at *p* ≤ 0.05 language: (including sentence repetition and verbal fluency).

**Table 4:** Multivariable logistic regression models evaluating factors associated with possible mild cognitive impairment (MoCA ≤ 25) (age and sex adjusted) (possible MCI (n = 61) vs.no possible MCI (*n* = 39).

	Model #1		Model #2	
	*P*	OR (LL – UL 95% C.I)	*p*	OR (LL – UL 95% C.I)
Sex [male]	**0.91**	0.94 (0.35–2.57)	**0.79**	1.15 (0.42–3.14)
Education [low (≤ 9 years)]	**< 0.001** ^*^	6.26 (2.30–17.02)	**0.001** ^*^	5.54 (2.08–14.81)
Age (years)	**0.70**	1.02 (0.92–1.14)	**0.86**	1.01 (0.90–1.13)
Plaque score	**0.02** ^*^	1.41 (1.06–1.86)		
ICA resistive index			**0.046** ^*^	1.87 (1.01–3.46)
Hosmer and Lemeshow test	χ^2^ = 7.49, *p* = 0.49		χ^2^ = 4.17, *p* = 0.84	

OR: Odd’s ratio C.I: Confidence interval LL: Lower limit UL: Upper Limit, MCI: MoCA screening cut-off for possible MCI: ≤25.

Model #1 ICA Resistive Index Excluded.

Model #2 Carotid Plaque Score Excluded.

Note: Resistive index and plaque score were not included together in the same model because they represent overlapping vascular measures.

Note: ICA RI was analyzed as a continuous variable; ORs represent each 0.1-unit increase.

*: Statistically significant at *p* ≤ 0.05.

## Discussion

In this cross-sectional study of community-dwelling older adults aged ≥ 60 years, we found that carotid atherosclerotic plaque burden, quantified by carotid plaque score, was independently associated with possible MCI. Even after adjusting for age, sex and education, higher plaque scores were associated with significantly greater odds of cognitive impairment. Additionally, increased ICA RI was associated with possible MCI, suggesting that both structural and hemodynamic markers of carotid disease are linked to cognitive performance. Domain-specific analyses further revealed that plaque burden was most strongly associated with impairments in visuospatial abilities and language performance. Together, these findings highlight a potential role for subclinical extracranial vascular pathology in domain-specific cognitive performance in aging populations.

Our findings are broadly consistent with a large body of evidence linking carotid atherosclerosis with cognitive dysfunction across diverse populations. Liang et al. reported in a cohort of 932 adults that the presence of ≥ 3 carotid plaques was significantly associated with poorer semantic fluency, and that greater intima–media thickness (IMT) was associated with reductions in global cognition and semantic fluency^12^. Although they did not observe a statistically significant association between plaque count and global cognition, their results reinforce the relevance of structural atherosclerosis to cognitive health in midlife and older age. Several population-based studies have reported similar results. In Ugandan older adults, plaque presence was strongly associated with cognitive impairment, while the Beaver Dam Offspring Study also showed associations between plaque scores and multiple cognitive domains dysfunction in middle-aged participants^13,14^. Our findings extend this evidence by demonstrating that plaque burden remains cognitively associated even in a narrower, older age group (≥ 60 years) using MoCA, a tool more sensitive to mild deficits than the MMSE used in several prior studies. The Tokyo Oldest Old Survey similarly identified lower MMSE performance among participants aged ≥ 85 years with high plaque scores, underscoring the predictive value of plaque burden in the oldest populations^15^. Our sample differed by including younger elderly adults, yet findings were directionally aligned. Longitudinal data from the Three-City Study and the BRAVE study also support these findings, showing that carotid plaque—but not IMT—predicts either dementia incidence or poorer cognitive performance^16,17^. Mechanistic insight is suggested by studies such as CABLE, where plaque presence correlated with amyloid and tau pathology, indicating a potential link between extracranial vascular disease and Alzheimer-related processes^18^. Similarly, Lin et al. demonstrated that advanced carotid atherosclerosis predicted poorer executive, visuospatial, and orientation performance over 10 years, mirroring the domain-specific associations we observed in visuospatial and language function^19^. Evidence from cardiovascular cohorts further supports the cognitive relevance of carotid pathology. Among patients with coronary heart disease, Molshatzki et al. found that high IMT and bilateral plaques were associated with lower cognitive performance even after excluding individuals with prior stroke^20^. A large Spanish cohort also demonstrated poorer cognitive performance across multiple domains in adults with carotid plaque, independent of stenosis severity^21^. Studies involving imaging modalities also confirm these observations. In the Framingham Offspring Study, even moderate internal carotid stenosis (≥ 25%) was associated with deficits in executive and nonverbal memory^22^. The ACE 1950 Study found a weak but significant association between plaque score and MoCA scores, similar to our findings, though multivariable adjustments weakened the association—possibly due to the younger study population^23^. Interestingly, the Northern Manhattan Study did not find significant associations between plaque presence or area and cognitive outcomes, although IMT was linked to episodic memory and plaques were associated with greater white-matter hyperintensity burden^24^. The authors noted important racial and ethnic heterogeneity that may influence vascular–cognition relationships. Overall, the consistency of evidence across continents, ethnicities, and study designs reinforces the biological plausibility of our findings.

We found that ICA RI was significantly higher in participants with possible MCI and remained independently associated with cognitive impairment in multivariate analyses. Higher RI reflects increased distal vascular resistance, suggesting impaired microvascular perfusion or reduced arterial compliance. This aligns with several previous studies. Rinjani et al. reported higher RI values among individuals with cerebral small vessel disease (CSVD) markers, and Wardlaw et al. demonstrated consistent associations between increased carotid pulsatility indices and poorer processing speed and visuospatial function—even when degree of stenosis was not predictive^25,26^. The ANTIQUE substudy corroborated these observations, showing that carotid plaque width, age, diabetes, and hypertension were key determinants of elevated RI^27^. Importantly, the threshold for abnormal RI (> 0.63) aligns closely with our findings, where our participants with possible MCI had a median RI of 0.63. Together, these findings suggest that hemodynamic compromise—independent of the degree of stenosis—may be linked to cognitive performance.

Several mechanisms plausibly explain the link between carotid atherosclerosis and cognitive dysfunction. Chronic cerebral hypoperfusion: Plaque-related arterial stiffening and reduced cerebrovascular reserve may lead to sustained reductions in cerebral blood flow, preferentially affecting watershed regions and fronto-subcortical networks that support executive and processing speed functions^28^. Microembolization and silent infarcts: Atheromatous plaques, particularly those with complex morphology, may shed microemboli causing subclinical infarcts that accumulate to impair cognition, especially in domains sensitive to distributed white matter integrity^29,30^. Impaired cerebrovascular reactivity: Elevated ICA RI and related indices indicate reduced autoregulatory capacity; impaired reactivity has been associated with domain-specific cognitive deficits and may potentiate vulnerability to additional vascular or neurodegenerative insults^31^. Systemic endothelial dysfunction and inflammation: Shared risk factors (hypertension, diabetes, dyslipidemia) promote both carotid atherogenesis and cerebral microvascular injury through endothelial dysfunction, oxidative stress and inflammatory pathways^32^. These pathways likely interact and vary in relative importance among individuals; hemodynamic compromise may dominate in some, whereas embolic or inflammatory mechanisms may be more relevant in others.

Educational attainment demonstrated the strongest independent association with possible MCI in both multivariable models. This finding is consistent with the well-recognized influence of education on cognitive reserve and performance on cognitive screening instruments such as the MoCA^33^. Evidence from Egyptian and international cohorts consistently demonstrates that educational attainment modifies cognitive trajectories across aging^34,35^. Interestingly, while education predicts baseline cognitive performance, its influence on rate of decline is less clear^36^; however, our findings support its robust protective effect on cognitive status in late life. Although educational level was adjusted for in the multivariable analyses and MoCA-Basic was used for participants with limited educational attainment according to the study protocol, residual confounding or some degree of misclassification cannot be excluded because cognitive impairment was defined using screening instruments rather than comprehensive clinical and neuropsychological assessment.

Key strengths of this study include: (1) the use of the Montreal Cognitive Assessment (MoCA), which is more sensitive than the Mini-Mental State Examination (MMSE) for detecting mild cognitive deficits; (2) the use of education-appropriate cognitive screening instruments, with the MoCA-Basic administered to participants with limited educational attainment or illiteracy to reduce the influence of education on cognitive screening performance; (3) quantitative carotid plaque scoring, allowing assessment of plaque burden rather than simple plaque presence or absence; (4) concurrent assessment of a hemodynamic marker (ICA RI), providing complementary structural and hemodynamic information; and (5) inclusion of community-dwelling Egyptian adults aged ≥ 60 years, an understudied population that expands the evidence on carotid atherosclerosis and cognitive function in Middle Eastern and North African populations.

Several limitations temper inference. First, the cross-sectional design precludes causal inference and temporal sequencing; plaque burden and elevated RI may be markers rather than drivers of cognitive decline. Second, cognitive impairment was classified using MoCA-based screening instruments rather than comprehensive clinical and neuropsychological assessment. Although education-appropriate cognitive screening instruments were used and educational attainment was adjusted for in the multivariable models, residual confounding related to education and cognitive reserve, as well as some degree of misclassification, cannot be excluded. Third, brain MRI was not routinely performed because the study included community-dwelling older adults without known neurological disease. Consequently, intracranial atherosclerosis, silent cerebrovascular lesions, and white matter changes could not be evaluated and may have influenced the observed associations. Fourth, single-center recruitment may limit generalizability to populations with different demographic or vascular risk profiles. Fifth, although ultrasound protocols were standardized, operator and measurement variability remain potential sources of error. From a research perspective, prospective longitudinal studies are warranted to determine whether progression of carotid plaque burden and persistent hemodynamic abnormalities are associated with subsequent cognitive decline and whether modification of vascular risk factors influences these associations.

## Conclusions

Carotid atherosclerotic plaque burden, rather than plaque presence alone, is independently associated with possible mild cognitive impairment in older adults, with elevated ICA resistive index providing complementary hemodynamic insight. Associations with visuospatial and language domains may reflect selective vulnerability to vascular pathology. These findings support carotid atherosclerosis—prior to significant stenosis—as a potentially clinically relevant marker associated with poorer cognitive performance. Incorporating carotid ultrasound into cognitive risk assessment may assist in identifying individuals who warrant further assessment. Longitudinal studies are needed to clarify causality, evaluate plaque progression, and assess whether vascular risk modification is linked to subsequent cognitive decline.

## Data Availability

The datasets generated and/or analyzed during the current study are available from the corresponding author on reasonable request.
